# Statistical and histological analysis of tumors of the upper extremity

**DOI:** 10.1007/s11678-015-0314-4

**Published:** 2015-05-11

**Authors:** Andrzej Zyluk, Agnieszka Mazur

**Affiliations:** Department of General and Hand Surgery, Pomeranian Medical University in Szczecin, ul. Unii Lubelskiej 1, 71-252 Szczecin, Poland

**Keywords:** Upper extremity benign tumors, Benign soft tissue tumors, Surgical management, Statistics, Histology, Benigne Tumoren der oberen Extremität, Benigne Weichteiltumoren, Chirurgische Versorgung, Statistik, Histologie

## Abstract

**Background:**

Tumors of the upper extremity are common and usually benign. The most commonly diagnosed are ganglion cysts: specific, non-neoplastic swellings localized mostly around the wrist.

**Objective:**

The objective of this retrospective study was to determine the proportion of various types of nonganglionic hand tumors operated on at the authors’ institution in 2014.

**Methods:**

A total of 246 patients, 141 women (57 %) and 105 men (43 %), with a mean age of 53 years and with tumorsof the upper extremity, were identified and treated in the authors’ institution in 2014.

**Results:**

Almost half of the lesions were localized in the fingers (*n* = 119, 48 %), followed by the wrist (*n*=49, 20 %), metacarpus (*n* = 40, 16 %), and more proximal parts (*n* = 38, 16 %). The time between the patients noticing the lesion and surgery was a mean of 4 years (range, 1 month to 30 years). The most common lesion was giant cell tumor of the tendon sheath (*n* = 58, 23 %), followed by lipoma (*n* = 40, 16 %), epidermal cyst (*n* = 23, 9 %), enchondroma (*n*=16, 6 %), hemangioma (*n* = 14, 6 %), fibroma (*n* = 11, (4 %), glomus tumor (*n* = 10, (4 %), and rheumatoid nodule (*n* = 10, (4 %). Two cases of malignant fibrosarcoma were identified incidentally.

**Conclusion:**

A brief review of the literature is made referring to the data presented here.

## Abstract (de)

**Hintergrund:**

Tumoren der oberen Extremität sind häufig und meistens gutartig. Die häufigsten Diagnosen betreffen Ganglionzysten: typische, nichtneoplastische Schwellungen, die zumeist im Bereich des Handgelenks lokalisiert sind.

**Ziel:**

Ziel der vorliegenden retrospektiven Studie war es, den Anteil der verschiedenen Typen von Handtumoren zu ermitteln, die keine Ganglionzyste darstellen und 2014 an der Klinik der Autoren operativ versorgt wurden.

**Methoden:**

Insgesamt wurden 246 Patienten, 141 Frauen (57 %) und 105 Männer (43 %), mit einem Durchschnittsalter von 53 Jahren und Tumoren der oberen Extremität erfasst und 2014 in der Klinik der Autoren behandelt.

**Ergebnisse:**

Fast die Hälfte der Läsionen waren an den Fingern lokalisiert (*n* = 119; 48 %), es folgten das Handgelenk (*n*=49; 20 %), die Mittelhand (*n* = 40; 16 %) und weiter proximal gelegene Körperteile (*n* = 38; 16 %). Die Zeitdauer zwischen Bemerkung der Veränderung durch den Patienten und der Operation betrug im Mittel 4 Jahre (Spannbreite: 1 Monat bis 30 Jahre). Die häufigste Veränderung war ein Riesenzelltumor der Sehnenscheide (*n* = 58; 23 %), es folgten Lipome (*n* = 40; 16 %), epidermale Zysten (*n* = 23; 9 %), Enchondrome (*n*=16; 6 %), Hämangiome (*n* = 14; 6 %), Fibrome (*n* = 11; (4 %), Glomustumoren (*n* = 10; 4 %) und Rheumaknoten (*n* = 10; 4 %). Als Zufallsbefund ergaben sich 2 Fälle eines malignen Fibrosarkoms.

**Schlussfolgerung:**

Abschließend wird eine kurze Übersicht über die Literatur zu den hier vorgestellten Daten gegeben.

## Introduction

Tumors of the hand and entire upper limb are frequent findings in hand surgeons’ practice. The hand is a complex structure, composed of several tissues, each of which can be the origin of proliferation and development of the tumor: skin, fat tissue, tendon sheath, muscle, nerve, bone, cartilage, etc. The most frequently encountered tumors of the upper extremity are listed in Table 1 and are grouped according to the tissue of origin. The most common is a ganglion cyst, which is a specific, non-neoplastic lesion arising either from the synovium of the joint or—less frequently—from the synovial sheath of the tendon. Ganglion cysts are typically located around the wrist and their diagnosis is usually establishedclinically. The reported incidence of particular types of tumors in the hand varies, but giant cell tumors of the tendon sheath, enchondromas, and lipomas are the most frequently mentioned [1–3]. Malignant tumors are relatively rare. The specific appearance and fast growth of the lesion may raise suspicion as to its malignant character.

**Table 1:** Histological categories of tumors of the upper extremity

Tissue or structure of origin	
**Soft tissue—benign**	
Skin	Epidermoid (sebaceous) cyst Wart (seborrheic wart, verruca vulgaris) Dermatofibroma Granuloma
Tendon sheath	Giant cell tumor of the tendon sheath Fibroma of the tendon sheath
Fat tissue	Lipoma Angiolipoma
Vessels	Hemangioma (simple and cavernous) Glomus tumor Hemangiopericytoma Vascular malformations Organized thrombus/hematoma
Nerves	Schwannoma Neurofibroma Plexiform neuroma
Muscles	Leiomyoma Angiomyoma
Fibrous tissue	Fibroma
Other	Rheumatoid nodules Gout nodules Synovitis
Bone	Enchondroma Giant cell bone tumor Osteoid osteoma Aneurysmal bone cyst Simple bone cyst Osteochondroma
**Malignant tumors**	
Skin	Basal cell carcinoma Squamous cell carcinoma Melanoma
Other soft tissue	Liposarcoma Myosarcoma Epithelioid sarcoma Eccrine adenocarcinoma Synovial cell sarcoma Malignant fibrous histiocytoma
Bone and cartilage	Chondrosarcoma Osteosarcoma Ewing sarcoma Metastatic tumors to the bones

Treatment of tumors in the hand is mostly surgical and consists in complete (radical) excision, as their character is not known preoperatively. It is usually performed in the operating theater, with the patient under adequate anesthesia, and in a bloodless operative field. All resected tumors are routinely sent for histological examination. Recurrences are not uncommon and are dependent on the character of the lesion, completeness of resection, and—to a certain degree—its localization. Removal of tumors arising from specific tissues, i.e., nerves, vessels, or bones, may require sophisticated techniques of reconstruction to avoid compromise of the hand function. In the case of malignant tumors that are preoperatively considered benign, secondary surgery is usually necessary (radicalization of the operation), followed by chemoradiotherapy.

The authors’ institution is the primary center of hand surgery in a part of the country having a population of about 4 million inhabitants, in which the vast majority of hand tumors are treated. The objective of this retrospective study was to investigate the prevalence, character, and demographic characteristics of all hand tumors operated on in 2013 and 2014.

## Patients and methods

The patients treated at our institution were mostly referred to us from surgical or family doctors’ outpatient clinics. Patients were included in this study if they met the following criteria: aged 18 years or older, with any soft tissue or bony tumor of the upper extremity, and a histological examination of the excised tumor being available. Exclusion criteria included suspected ganglion cyst, foreign body, arthritis deformity, or osteophyte and high suspicion of malignant tumor (skin carcinoma, melanoma). In all, 246 patients, 74 % of a total of 334 who were operated on for upper extremity tumors in 2014 year, met the inclusion criteria. Histological examination of the tumors was performed in the Department of Pathology at the Pomeranian Medical University in Szczecin. The study was retrospective and the following data were analyzed from the institutional database: age and sex of the patients, tumor location, X-ray examination (if performed), disease duration, and results of the histological examination of the tumor.

## Results

A total of 246 patients, 141 women (57 %) and 105 men (43 %), aged a mean of 53 years were included. Duration of the disease (time between the patients noticing the lesion and surgery) was a mean of 4 years (range, 1 month to 30 years). The location of the tumors is shown in Table 2. Almost half of the lesions were localized in the digits, followed by the wrist, metacarpus, and more proximal parts of the limb. The most often-occurring lesion type was giant cell tumor of the tendon sheath, followed by lipoma, epidermal cyst, enchondroma, and hemangioma (Table 3). Soft tissue swellings were much more common than bony lesions (94 vs. 6 %).

**Table 2:** Localization of tumors in the upper extremity

Localization	Number of cases	%
Digits	119	48
Wrist	49	20
Metacarpus	40	16
Forearm	25	10
Arm	13	6

**Table 3:** Incidence of tumors identified in the study of 246 patients

Tumor type	Number of cases	%
Giant cell tumor of the tendon sheath	58	23
Lipoma	40	16
Epidermoid cyst	23	9
Enchondroma	16	6
Hemangioma	14	6
Glomus tumor	11	4
Fibroma	10	4
Rheumatoid nodule	10	4
Schwannoma	7	2.8
Gout nodule	7	2.8
Wart (seborrheic)	6	2.4
Glomangioma	6	2.4
Keratopapilloma	6	2.4
Granuloma telangiectasia	5	2
Synovitis	5	2
Neurofibroma	4	1.6
Organized thrombus	4	1.6
Vascular malformation	3	1.2
Malignant tumors	2	0.8
Other (single)	9	3.6
Total	246	100

### Location of tumors

Giant cell tumors of the tendon sheath (*n* = 58) were localized most commonly on the dorsal (*n* = 25, 43 %) or palmar side of the digits (*n* = 17, 29 %), followed by the wrist (*n* = 9, 16 %) and the palmar metacarpus (*n* = 7, 12 %). Lipomas (*n* = 40) were more frequently seen in the forearm and arm (*n* = 29, 73 %) than in the hand or wrist (*n* = 11, 27 %). Two tumors were located deep in the thenar muscles and one in the deep palmar space. Of the epidermoid cysts (*n* = 23), the majority was seen in the skin of the dorsal aspect of the digits and metacarpus (*n* = 15), and the remaining eight in the wrist and forearm. Enchondromas (*n* = 16) were most frequently seen in the digits, in the proximal (*n* = 9), middle (*n* = 5), and distal phalanges (*n* = 2). Most of them involved the ring and little fingers (*n* = 10). Hemangiomas were located mostly on the palmar side of the fingers (*n* = 8), in the wrist (*n* = 3), forearm (*n* = 2), and on the palm (*n* = 1).

### Malignant tumors

In two cases, excised tumors appeared to be malignancies. Both were fibrosarcoma: one mimicking (resembling) a ganglion cyst localized in the wrist, and one with a presentation similar to that of a cystic tumor on the dorsum of the hand. In both cases primary surgery was limited to excision of the lesion, but after diagnosis of the malignancies both patients underwent radicalization of the procedure (extended excision of the scar with oncological margins).

## Discussion

Tumors of the hand and entire upper limb are frequent in hand surgeons’ practice. Their presentation, diagnosis, and treatment may be different than for similar lesions affecting other parts of the body, mostly because of the complex anatomy and functional characteristics of the hand [1]. There is discrepancy in the literature regarding the incidence of soft tissue and bony tumors of the upper extremity. In a majority of the studies (including our own), soft tissue lesions had the highest incidence, while some authors report enchondromas to be the most common [4]. This is probably a result of the different patient selection criteria used in the studies, i.e., a proportion of minor soft tissue lesions may be operated on in outpatient settings and thus not be recorded in clinical databases. Management of bone tumors almost always requires hospital admission.

The most common pathology in our series was giant cell tumor of the tendon sheath. This finding is consistent with results reported in the literature [1–3]. Giant cell tumors of the tendon sheath usually present as slow-growing lesions localized mostly on the digits, adjacent to the interphalangeal joints (in our series more common on the dorsal side of the digits). In most cases they are asymptomatic, but in some patients they may cause mild pain and discomfort or may interfere with hand function. Histologically, giant cell tumor is composed of multinucleated giant cells, histiocytes, fibrotic material, and deposits of hemosiderin [1]. Except for soft tissue involvement, giant cell tumors are found in bones, with the most common location in the epiphyses [1, 2]. Treatment of these tumors consists in local excision. The recurrence rate reported in the literature is relatively high (a mean of 15 %, range 4–40 %) and is mostly caused by incomplete excision of the tumor and by overlooking residual satellite nodules. Giant cell tumor of the tendon sheath is poorly encapsulated and frequently involves adjacent bones, joints, or tendons, which makes the radical excision difficult to perform. Satellite nodules are also relatively common.

**Fig. 1:**
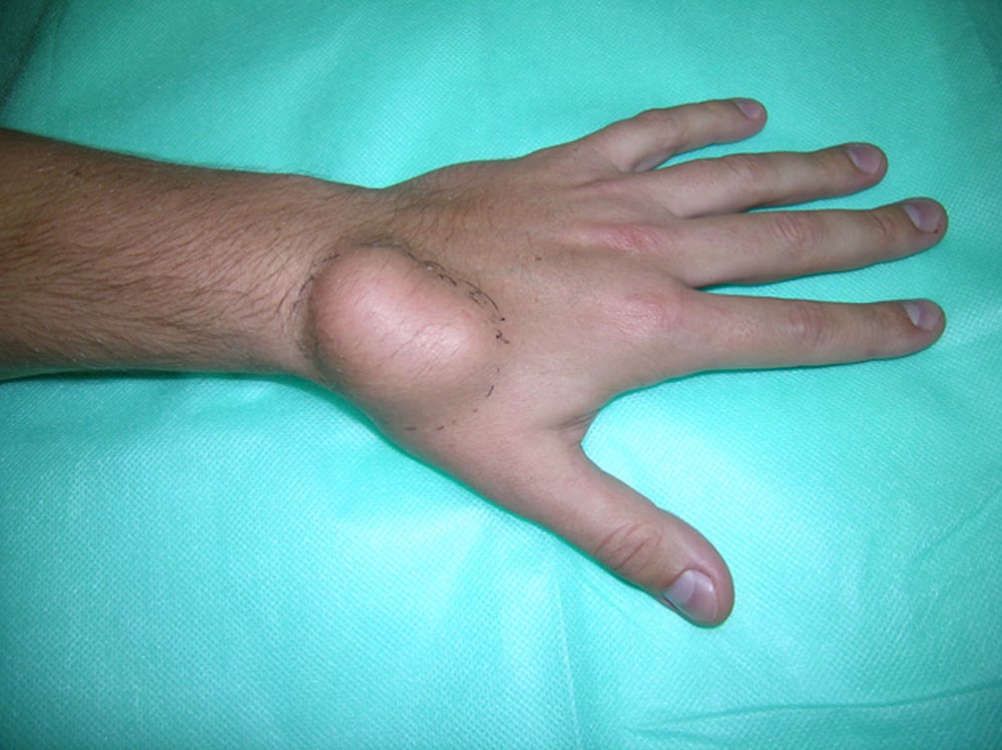
Lipoma on the dorsum of the hand

**Fig. 2:**
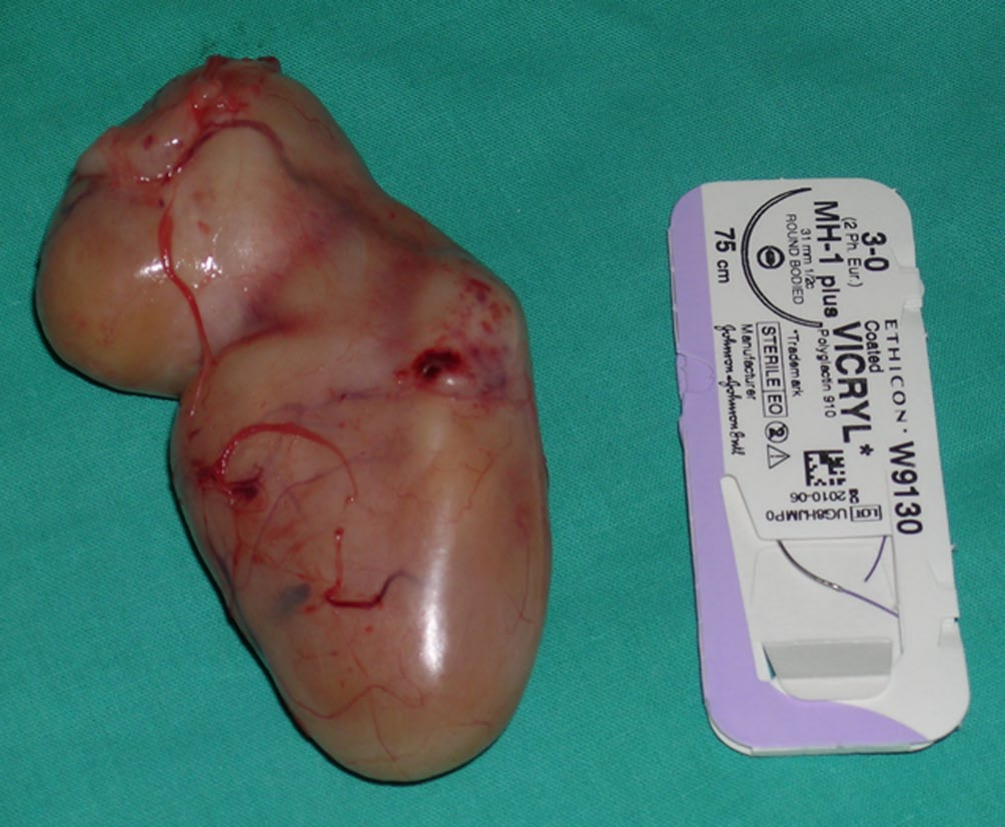
View of a resected lipoma

**Fig. 3:**
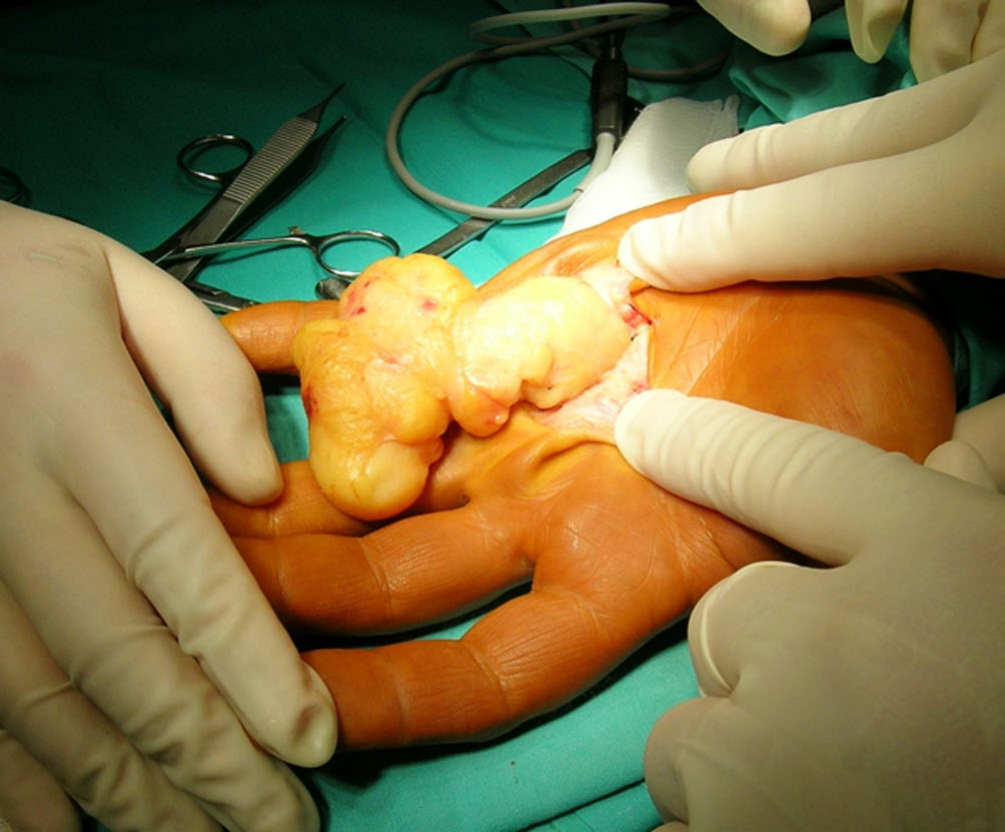
Retrieval of lipoma located deep in the hand

Lipomas were the second commonest pathologies in our series. These tumors have no typical location and may be found anywhere in the upper extremity. In our series they were more frequently seen in the forearm and arm than in the hand or wrist (29 vs. 11 cases). Lipomas typically presented as soft, mobile, and painless masses, relatively easy to diagnose correctly before operation. They are mostly asymptomatic, but may cause complaints if present near the nerves (i.e., in the carpal tunnel) [4]. If localized superficially they are easy to excise, as the majority has a well-defined capsule (Figs. 1 and 2). In a deeper location, i.e., in the palmar space (Fig. 3), carpal tunnel, or intramuscularly, they may be technically more demanding to remove. The risk of malignant transformation is rare, but it increases with the size of the tumor. The recurrence rate after excision is low, less than 5 % [2].

Epidermoid (sebaceous) cysts were the third most common in our series. They typically present as soft, immobile, and painless lumps under the skin. Most of these cysts have a well-defined capsule filled with a cheese-like, fatty substance, secreted by the cellular layer of the capsule. Epidermoid cysts most often arise from swollen hair follicles, and thus are not seen on the palm. Treatment consists in excision of the entire capsule, but recurrences are not uncommon. Malignant transformation is rare, but some skin cancers may present clinically as epidermoid cysts [2].

Bony lesion, enchondroma, was the fourth most frequent pathology in our series. This is the most common bone tumor reported in the literature, constituting about 90 % of all osteogenic lesions encountered in the hand [5–7]. Although commonly considered as a bony lesion, enchondroma probably arises from aberrant cartilaginous foci, thus it should be classified as a cartilage tumor [5, 7]. In the upper extremity the lesion is most commonly located in the proximal and middle phalanges, followed by the metacarpal bones and the distal radius. In our series they were seen only in the digits (more commonly in two ulnar fingers) and in the proximal phalanges. Enchondroma is usually asymptomatic and is relatively frequently diagnosed when it causes pathological fracture of the phalanx, as occurred in seven patients in our series. The diagnosis is based on radiological examination, showing typically well-circumscribed, cystic-like, radiolucent lesion, located in the periarticular part of the phalanx. Although benign, enchondromas are locally destructive and may invade adjacent structures, such as joints, tendons, and nerves. Management of small enchondromas includes curettage of the lesion alone, but in larger tumors the curetted cavity is filled with cancellous bone graft or synthetic substances (polymethylmethacrylate, calcium phosphate bone cement). In our series, half of the patients underwent curettage alone, whereas the second half received cancellous bone graft harvested from the distal radius. Pathologic fractures underwent primary treatment (conservative or operative with K-wire pinning) and tumor curettage was delayed until the fracture healed. This schedule is consistent with the literature reports showing a higher rate of complications in patients undergoing tumor excision before fracture consolidation [7, 8]. Malignant transformation of enchondromas is uncommon, except of multiple endochondromatosis as in the case of Ollier disease and Maffucci syndrome.

**Fig. 4:**
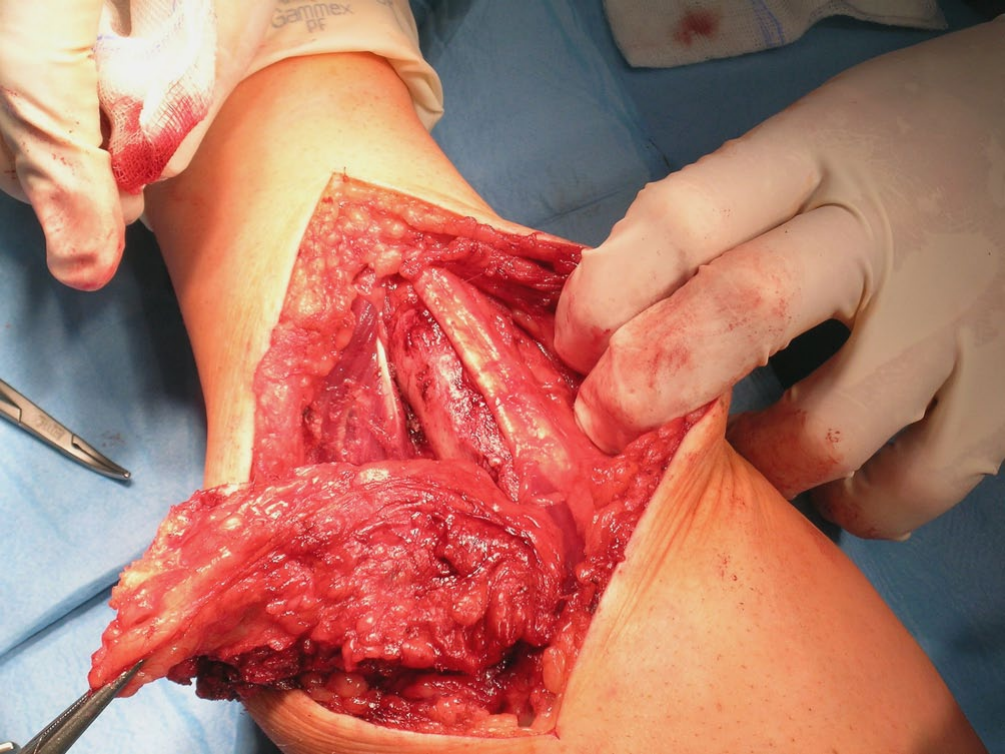
Cavernous hemangioma resected in the forearm

**Fig. 5:**
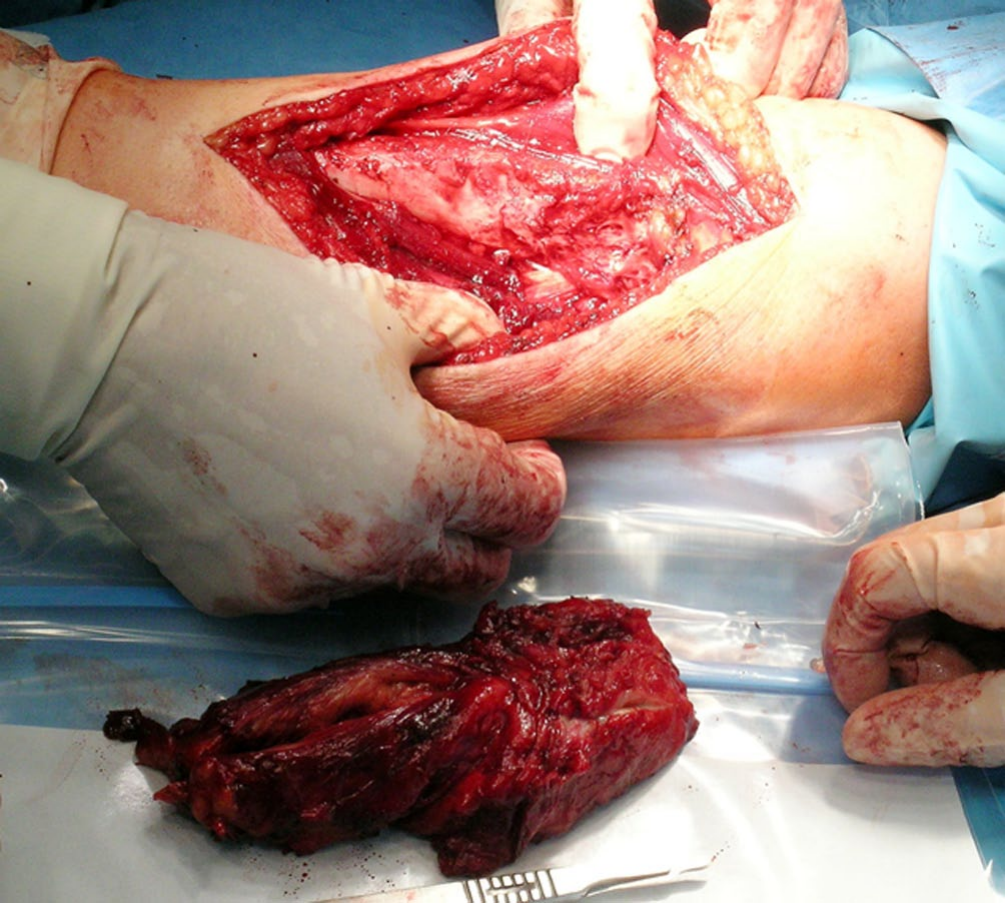
View of the operated site after removal of the hemangioma and the tumor

Hemangiomas were the fifth most frequent tumor type in our series. They are more common in children, but are also seen in young adults, as was the case in our sample (mean age of patients, 22 years; range, 18–31 years). Hemangioma typically presented as a soft, reddish-cyanotic mass, painless in most cases. It occurred mostly on the palmar surface of the digits (in 8 of 14 cases). In two patients the lesion was deeply localized in the forearm muscles, causing pain and swelling of the forearm. The diagnosis in these cases (cavernous hemangioma) was made based on magnetic resonance imaging studies. Treatment of hemangiomas consists in surgical excision, sometimes by coverage of the defect with skin graft or local flap. The operation may be technically demanding and precautions should be taken to avoid vascular compromising of the digits. Cavernous hemangiomas localized deeply in the muscles are also difficult to remove, as they may be extensive and infiltrate muscles without a distinct margin between normal and pathological tissue (Figs. 4 and 5).

**Fig. 6:**
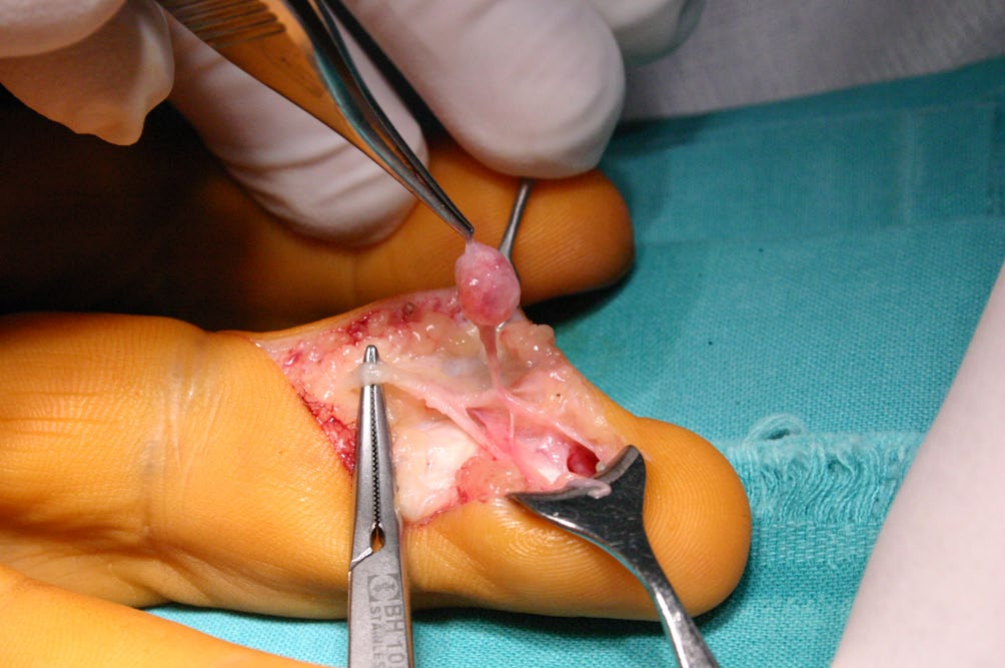
Glomus tumor localized in the pulp of the finger

Glomus tumors were also diagnosed in our series (Fig. 6). Glomus tumor is a benign hamartoma arising from a neuromyoarterial structure called a glomus body, which controls blood pressure and temperature. This lesion can be located anywhere, but the subungual region of the distal phalanx is particularly frequent, as occurred in all our cases. Glomus tumor presents as a tender part of the nail, and the painful area (or point) can be precisely determined by the patient. Local pain and tenderness are typically increased by exposure to cold. Radiographs may show erosion or a cavity of the bone of the distal phalanx, adjacent to the tumor. Nail plate deformity may also be seen. The diagnosis is relatively easy and surgical excision is usually curative with a low rate of recurrence [2, 9].

**Fig. 7:**
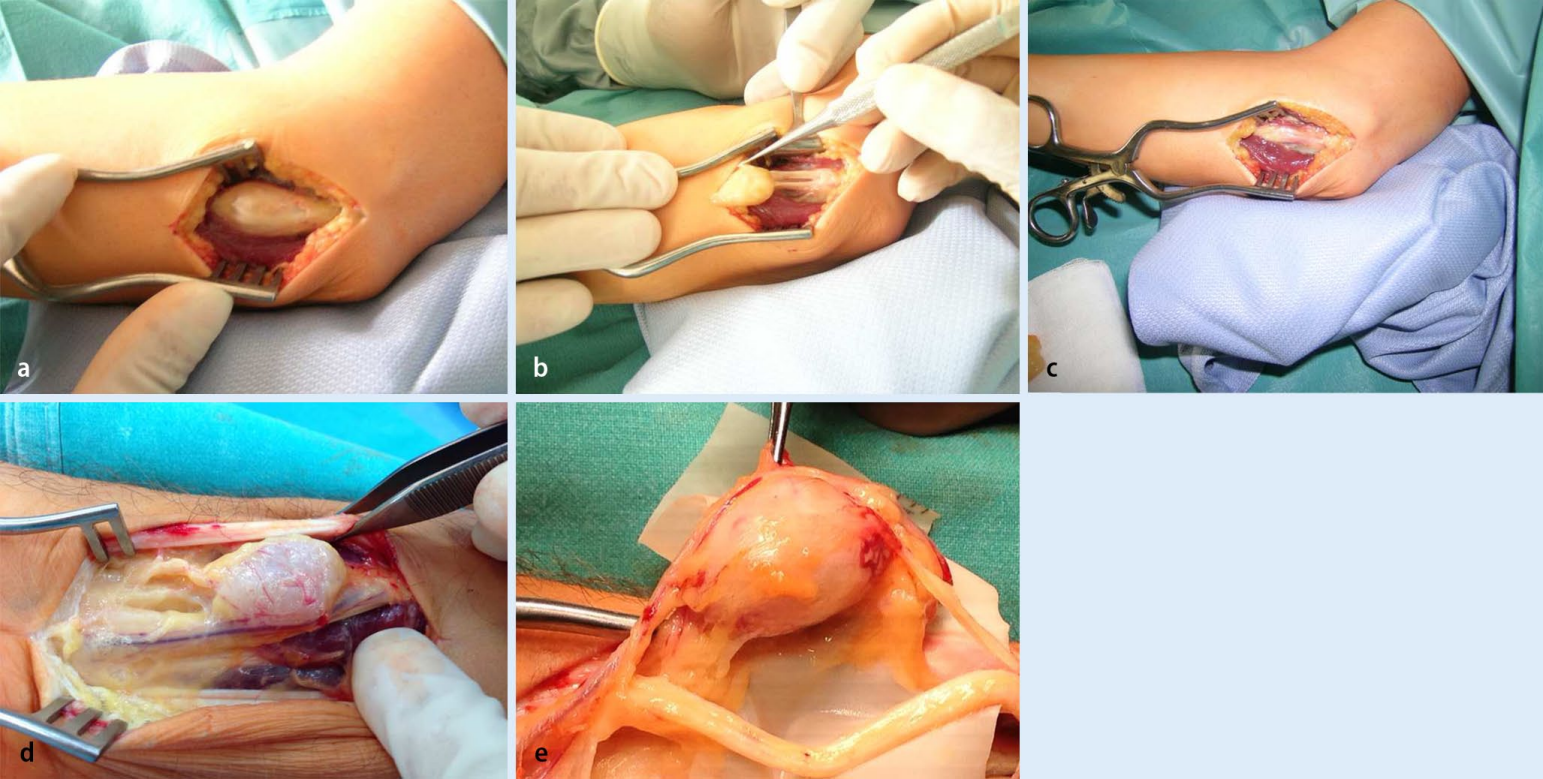
**a** Schwannoma of the ulnar nerve found incidentally in a patient operated on for cubital tunnel syndrome. **b** Enucleation of the tumor from the ulnar nerve. **c** View of the nerve after removal of the tumor. **d**, **e** Schwannoma of the median nerve

Other tumors were less common. Relatively interesting were nerve tumors, all of which were schwannomas (*n* = 7). Four of them were localized in the median nerve, two in the ulnar nerve on the forearm, and one arose from the digital nerve on the index finger. Five of these tumors were preoperatively diagnosed as involving the nerve, but two were found incidentally during operation. In all cases, tumors were enucleated from the nerve without compromising nerve structure and function (Figs. 7a, b, c, d, and e).

Two malignancies were diagnosed postoperatively among 246 excised tumors (0.8 %). In both cases, at presentation the lesion looked innocent and was considered benign. This finding confirms the necessity of a histological examination of most tumors after removal, except for typical-looking ganglia. Doubts may arise when treating patients with asymptomatic, slow-growing soft tissue tumors that are thought to be benign. We are fairy frequently faced with this problem in our ambulatory practice. Most of these patients do not wish to have surgery and observation is one of the accepted options in these cases. Additional imaging (i.e., ultrasonography or magnetic resonance imaging) may make the diagnosis more accurate, but is associated with higher costs and is never 100 % reliable. Excisional biopsy of a tumor in the finger or wrist does not differ from its definitive removal, and is not recommended in small lesions. Sluijmer et al. suggested that the hand surgeon’s preoperative diagnosis without imaging is usually correct prior to excision of a mass in the hand. Discrepant diagnoses are usually benign and do not alter treatment [3]. These findings support observation as a reasonable option in innocent-looking, slow-growing tumors of the hand; however, this approach is not commonly accepted.
